# Effect of knockout of α_2_δ-1 on action potentials in mouse sensory neurons

**DOI:** 10.1098/rstb.2015.0430

**Published:** 2016-08-05

**Authors:** Wojciech Margas, Laurent Ferron, Manuela Nieto-Rostro, Arnold Schwartz, Annette C. Dolphin

**Affiliations:** 1 Department of Neuroscience, Physiology and Pharmacology, University College London, London WC1E 6BT, UK; 2 College of Medicine, University of Cincinnati, Cincinnati, OH 45267-0557, USA

**Keywords:** calcium channel, auxiliary subunit, action potential, calcium transient, excitability

## Abstract

Gene deletion of the voltage-gated calcium channel auxiliary subunit α_2_δ-1 has been shown previously to have a cardiovascular phenotype, and a reduction in mechano- and cold sensitivity, coupled with delayed development of neuropathic allodynia. We have also previously shown that dorsal root ganglion (DRG) neuron calcium channel currents were significantly reduced in α_2_δ-1 knockout mice. To extend our findings in these sensory neurons, we have examined here the properties of action potentials (APs) in DRG neurons from α_2_δ-1 knockout mice in comparison to their wild-type (WT) littermates, in order to dissect how the calcium channels that are affected by α_2_δ-1 knockout are involved in setting the duration of individual APs and their firing frequency. Our main findings are that there is reduced Ca^2+^ entry on single AP stimulation, particularly in the axon proximal segment, reduced AP duration and reduced firing frequency to a 400 ms stimulation in α_2_δ-1 knockout neurons, consistent with the expected role of voltage-gated calcium channels in these events. Furthermore, lower intracellular Ca^2+^ buffering also resulted in reduced AP duration, and a lower frequency of AP firing in WT neurons, mimicking the effect of α_2_δ-1 knockout. By contrast, we did not obtain any consistent evidence for the involvement of Ca^2+^-activation of large conductance calcium-activated potassium (BK) and small conductance calcium-activated potassium (SK) channels in these events. In conclusion, the reduced Ca^2+^ elevation as a result of single AP stimulation is likely to result from the reduced duration of the AP in α_2_δ-1 knockout sensory neurons.

This article is part of the themed issue ‘Evolution brings Ca^2+^ and ATP together to control life and death’.

## 1. Introduction

One of the main tasks of neurons is propagation and processing of information, which is encoded in the form of action potentials (APs). APs are initiated by depolarization of the cell membrane, and inflow of Na^+^ through voltage-gated sodium channels [1]. They are terminated by inactivation of these channels, as well as activation of potassium channels and consequent repolarization of the cell membrane. The kinetic and voltage-dependent properties of all the channels involved, including activation, inactivation and reactivation, are well tuned, and slight changes of kinetic parameters can affect AP duration and frequency of firing in each cell. During an AP, the opening of voltage-gated calcium (Ca_V_) channels (VGCCs) is also triggered, which results in Ca^2+^ influx, particularly during the falling phase of the AP. Ca^2+^ entry leads to short- and long-term cellular events, including release of neurotransmitters, modulation of neuronal excitability, initiation of phosphorylation cascades and regulation of gene expression [2].

VGCCs are made up of a pore-forming α_1_ subunit, associated—except in the case of T-type channels—with auxiliary β and α_2_δ subunits [3]. In addition, a γ1 subunit has been found associated with skeletal muscle α_1_ subunits [4]. The β subunit protects the channels from proteasomal degradation and acts as a chaperone protein [5,6]. It binds to the cytoplasmic I–II loop to promote proper folding of the S6 segment of domain I, and increases surface expression of the channels [3,7,8]. The β subunit also modulates the gating properties of the α1 subunit; it shifts channel activation to more negative potentials and increases the open probability of the channels, thus increasing the macroscopic currents of recorded Ca_V_1 and Ca_V_2 channels by several mechanisms [3].

The α_2_δ accessory subunits are membrane-associated extracellular proteins that markedly increase Ca^2+^ current density for the Ca_V_1 and Ca_V_2 channels, in part by increasing cell surface expression of the channels [8–10], and also by decreasing channel turnover, as inferred from radiolabelled conotoxin binding [11]. They also increase the inactivation rate of Ca_V_1 and Ca_V_2 channels, and hyperpolarize the steady-state inactivation of most Ca_V_1 and Ca_V_2 channels tested (for review, see [12]). Furthermore, overexpression of α_2_δ-1 in hippocampal neurons resulted in increased neurotransmitter release in response to a single AP [13], which was hypothesized to be a result of a change in calcium channel distribution in active zones. It was further observed that α_2_δ-1 overexpression resulted in shorter AP duration in dorsal root ganglion (DRG) neuron somata and also reduced presynaptic AP duration in hippocampal synaptic boutons [13,14].

Peripheral nerve injury results in upregulation of α_2_δ-1 in damaged DRG neurons, and the development of behavioural hypersensitivity to sensory stimuli in the affected limb [15,16]. Furthermore, in transgenic mice constitutively over-expressing the α_2_δ-1 subunit, there was a lowered threshold for response to mechanical and thermal stimuli, comparable to the level of allodynia and hyperalgesia manifested by animals that underwent nerve injury [17]. Behavioural sensitization was accompanied by changes at the molecular level, as small DRG neurons isolated from α_2_δ-1 over-expressing mice had larger calcium currents, and more rapid activation rate than their wild-type (WT) counterparts.

The α_2_δ-1 knockout (KO) mice used in this study result from disruption of the α_2_δ-1 gene with a targeted insertion in exon 2 [18]. These animals were found to have a minor cardiovascular phenotype, and the L-type calcium currents recorded from isolated cardiomyocytes were significantly reduced, as well as showing a shift to positive potentials of the steady-state activation and inactivation curves, as a result of the ablation of α_2_δ-1 [18]. We then found a marked reduction in baseline mechanosensitivity in α_2_δ-1 KO mice, and a striking delay in the development of neuropathic hypersensitivity following nerve injury [19]. Furthermore, calcium channel currents recorded in DRG neurons from α_2_δ-1 KO mice showed significantly reduced density [19].

In this study, we therefore explored the effects of α_2_δ-1 KO on the excitability of the DRG neurons of these mice, in order to dissect how the calcium channels that are affected by α_2_δ-1 KO are involved in setting the duration of individual APs and their firing frequency in DRG neurons.

## 2. Material and methods

### (a) Mice

Heterozygotes from the α_2_δ-1 knockout mice described previously [18] were obtained from the laboratory of Dr Arnold Schwartz on a Black Swiss/C57Bl/6 background, and re-derived at the Mammalian Genetics Unit Harwell, UK, using sperm from imported *cacna2d1*^+/−^ males to fertilize WT C57BL/6 J egg cells, before implanting into pseudo-pregnant female mice. They were further backcrossed onto the C57Bl/6 J background before use in our previous study [19].

### (b) Cell isolation

DRGs were dissected from the spine of WT and α_2_δ-1 KO littermate mice of both sexes, as stated, aged 9–15 weeks (11.5 ± 3.5 weeks, *N* = 62 mice, for electrophysiological experiments; 10.2 weeks, *N* = 6 mice for Ca^2+^ imaging experiments). All dissections and experiments were performed with the experimenter blind to the genotype. Cell cultures were obtained after enzymatic and mechanical dispersal as described previously [20]. Briefly, ganglia were incubated in HBSS containing 100 U ml^−1^ DNase, 5 mg ml^−1^ dispase, and 2 mg ml^−1^ collagenase type 1A for 30 min at 37°C, and dissociated DRG neurons were plated on poly-l-lysine-coated glass coverslips and maintained in DMEM/F12 supplemented with 10% FBS, 2 mM GlutaMAX, penicillin 100 U ml^−1^, streptomycin 100 µg ml^−1^ (Invitrogen).

### (c) GCaMP imaging in DRG neurons

DRG neurons were transfected with pCAGGs-mCherry and membrane-directed pGPCMV-GCaMP6s-CAAX [21] (obtained from Addgene), in a ratio of 1 : 3 using an Amaxa Nucleofector (Lonza) as previously described [22]. After 4–5 days in culture, coverslips were mounted in a laminar-flow perfusion and stimulation chamber (Warner Instruments) on the stage of an epifluorescence microscope (Axiovert 200 M, Zeiss). A blue (470 nm emission peak) light-emitting diode served as the light source (Cairn Research, UK) and fluorescence excitation and collection were done through a 40 × 1.3 NA Fluar Zeiss objective using 450/50 nm excitation and 510/50 nm emission and 480 nm dichroic filters. Live cell images were acquired as previously described with minor modifications [22]. GCaMP fluorescence was collected at 500 Hz over a 512 × 35 pixel area. Cells were perfused (0.5 ml min^−1^) in a saline solution at 22°C containing (in millimolar) 119 NaCl, 2.5 KCl, 2 CaCl_2_, 2 MgCl_2_, 25 HEPES (buffered to pH 7.4), 30 glucose. Neurons were stimulated by passing 1 ms current pulses through the field stimulation chamber via platinum electrodes. Neurons expressing GCaMP6s-CAAX were identified by first stimulating the preparation at 33 Hz for 180 ms every 4 s. Subsequently, single stimulations of 1 ms (mimicking single AP) were followed by a delay of 1 s and then a 100 Hz stimulation for 1 s to outline the processes of the neurons. Analysis was performed with ImageJ (http://rsb.info.nih.gov/ij), using a custom-written plugin (http://rsb.info.nih.gov/ij/plugins/time-series.html). Regions of interest (ROI, 2 µm diameter circles) in the somata were placed adjacent to the plasma membrane. ROI in the main axonal processes were selected between 10 and 20 µm away from the cell body.

### (d) Electrophysiological recordings

DRG neurons were used for electrophysiological experiments after 4–5 days in culture, and had extensive neurites. Excitability was assessed in the whole-cell current-clamp configuration, recorded at 22°C. Borosilicate glass electrodes (Plowden & Thompson Ltd, UK) were pulled with a micropipette puller (P-97 Pipette Puller, Sutter Instruments, CA, USA) and fire-polished with a microforge (MF-83 Microforge, Narishige, Japan) to obtain resistances of the electrodes in the bath solution between 1 and 4 MOhm. Recordings were made with an Axopatch 200A amplifier (Axon Instruments, Burlingame, CA, USA), at a sampling frequency 20 kHz, low-pass filtered at 10 kHz with a built-in 8-pole Bessel filter; the signal was analogue/digital converted by a Digidata 1322A (Axon Instruments), and data were collected with pClamp 9.2 software (Axon Instruments), after filtering with a 1 kHz digital 8-pole Bessel filter.

In all experiments, recordings were started in voltage-clamp configuration. The holding potential (HP) was set to −70 mV, the cell membrane capacitance (*C*_m_) was estimated by pClamp9 and series resistance was read directly from the amplifier and compensated at 80%. To record AP activity, the amplifier was switched to current-clamp gap-free recording mode with no biased current applied, so the resting membrane potential (RMP) could be measured. Next, biased current was injected to change the HP to −60 or −70 mV (as stated) from which voltage–current (V–I) relations were recorded. AP activity was elicited by injection of a series of 400 ms current pulses every 5 s, starting from −10 pA, followed by currents injected in increasing amplitude every +10 pA, until AP discharge rate reached maximum frequency. Input resistance (*R*_in_) was determined as the ratio of the steady-state membrane potential estimated with a single component exponential function fit to the rate of voltage change evoked by the −10 pA step current injected.

The external recording solution contained (in millimolar): NaCl 145, KCl 5, CaCl_2_ 2, MgSO_4_ 1, HEPES 10, Glucose 10. The pH was adjusted to 7.4 with 1 M NaOH, osmolarity was measured and adjusted with sucrose to 10 mOsm more than in the pipette solution. The intracellular free [Ca^2+^] was calculated to be 39.5 nM (http://maxchelator.stanford.edu/CaMgATPEGTA-TS.htm). The standard pipette solution used for most recordings contained (in millimolar): KCl 130, EGTA 10, HEPES 10, NaCl 8, Mg-ATP 4, MgCl_2_ 1, CaCl_2_ 1, Na_2_-GTP 0.4, osmolarity was 310 mOsm, pH 7.2 adjusted with 1 M KOH. In experiments designed to block SK channels with the specific blocker apamin, the pipette solution contained a reduced concentration of calcium chelator EGTA, and reduced intracellular [Ca^2+^], to maintain the basal intracellular free [Ca^2+^] at 39 nM, but enable activation of SK channels, by reducing Ca^2+^ buffering. The solution contained (in millimolar): KCl 137, EGTA 1, HEPES 10, NaCl 8, Mg-ATP 4, MgCl_2_ 1, CaCl_2_ 0.1, Na_2_-GTP 0.4 [23]. In all electrophysiological recordings performed with the standard pipette solution only DRG neurons isolated from male mice were used, while cells isolated from both male and female mice were used in experiments using the reduced Ca^2+^-buffering pipette solution. No differences were observed between data from male and female mice.

### (e) Potassium channel blockers

Drugs used were 0.1 µM iberiotoxin (Alomone Labs, Israel) and 0.5 µM apamin (Alomone). Working solutions containing these drugs were freshly made from stock solutions just before each experiment and were applied by a custom-made gravity-fed perfusion system, with a 250 µm internal diameter tube positioned about 300 µm from the recorded cell. When applying drugs in current-clamp mode, cells were recorded in continuous recording mode for 1 min before starting the V–I protocol. Initial experiments showed that 1 min of exposure to these blockers is sufficient to elicit the full effect.

### (f) Statistical analysis

The frequency of AP firing was analysed with Clampfit 10.2 (Axon Instruments), the % duration of APs and all statistical tests were assessed by GraphPad Prism 4.0 (GraphPad Software, La Jolla, CA, USA), and graphs were plotted with Origin 7 (Origin Lab, Northampton, MA, USA). Data are presented as mean ± s.e.m., *n* indicates number of cells recorded. To determine statistical difference between two groups, Student's *t*-test or paired *t*-test was applied as appropriate, otherwise to determine the difference between more than two groups one- or two-way ANOVA analysis was performed whenever appropriate, followed by multiple *post hoc* group comparison when the overall *p*-value for the ANOVA was less than 0.05.

## 3. Results

### (a) Comparison of intracellular Ca^2+^ elevation resulting from a single AP stimulation in WT and α_2_δ-1 KO DRG neurons

**Figure 1.**
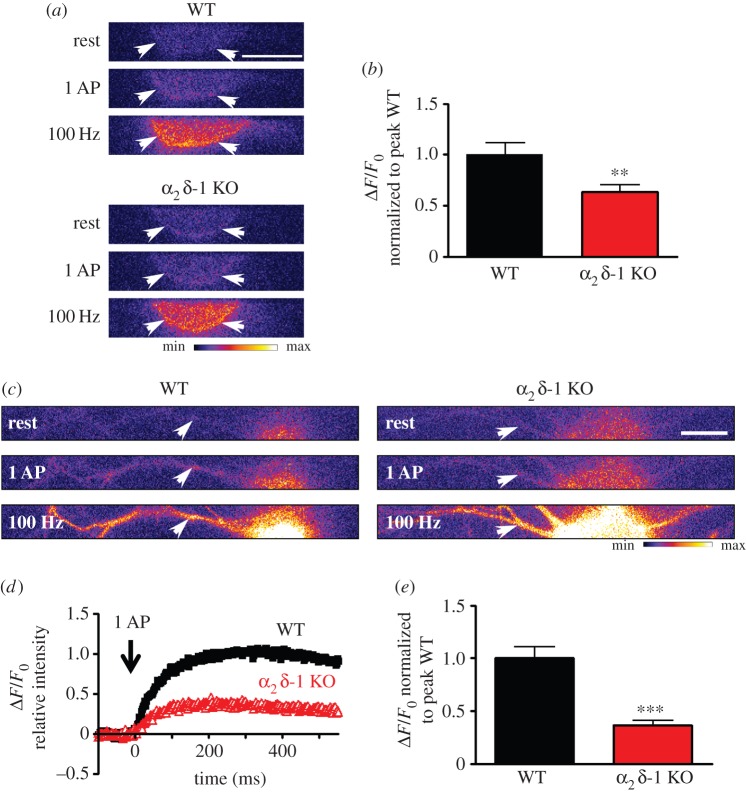
Effect of α_2_δ-1 KO on Ca^2+^ influx into the soma and proximal segment of DRG neurons. (*a*) Imaging GCaMP6s activity in cell bodies from WT (upper three panels) and α_2_δ-1 KO (lower three panels) DRG neurons, in response to electrical stimulation. White arrows indicate the region of the soma used to quantify GCaMP6s variations. Top panels: at rest; middle panels: after 1 AP; bottom panels: after 100 Hz stimulation for 1 s. Scale bar, 20 µm. The pseudocolour scale is shown below the panels. (*b*) Average peak GCaMP6s intensity in response to 1 AP. GCaMP6s intensity was normalized to the peak value in WT condition. WT (black bar): 1.0 ± 0.12 (*n* = 57); *α*_2_*δ*-1 KO (red bar): 0.64 ± 0.07 (*n* = 56), **p = 0.0083, Student's *t*-test. Average peak GCaMP6s intensity after 1 s at 100 Hz (normalized to the peak value for 1 AP in WT condition): WT 26.9 ± 3.3 (*n* = 58); α_2_δ-1 KO 22.7 ± 2.4 (*n* = 56), *p* = 0.31. (*c*) Imaging GCaMP6s activity in processes from WT (left three panels) and α_2_δ-1 KO (right three panels) DRG neurons, in response to electrical stimulation. White arrows indicate the regions of the processes used to quantify GCaMP6s variations. Top panels: at rest; middle panels: after 1 AP; bottom panels: after 100 Hz stimulation for 1 s. Scale bar, 20 µm. The pseudocolour scale is shown below the right panel. (*d*) Average time course of Ca^2+^ influx in response to 1 AP in processes of WT (black squares) and α_2_δ-1 KO (red open triangles) DRG neurons. (*e*) Average peak GCaMP6s intensity in response to 1 AP. GCaMP6s intensity was normalized to the peak value in WT condition. WT (black bar): 1.0 ± 0.10 (*n* = 71); α_2_δ-1 KO (red bar): 0.37 ± 0.04 (*n* = 76), ***p < 0.0001, Student's *t*-test. Average peak GCaMP6s intensity after 1 s at 100 Hz (normalized to the peak value for 1 AP in WT condition): WT 16.6 ± 2.6 (*n* = 72); α_2_δ-1 KO 14.2 ± 1.9 (*n* = 76), *p* = 0.45.

Building on our previous findings that cultured DRG neurons from α_2_δ-1 KO mice had reduced calcium current density *in vitro*, and reduced excitability *in vivo* [19], we then investigated intracellular Ca^2+^ responses to a single AP (figure 1). We concentrated on small–medium DRG neurons, as these neurons express α_2_δ-1 to a greater extent than larger DRGs [15]. DRG neurons were transfected with a genetically engineered Ca^2+^ indicator GCaMP6s, which is targeted to the plasma membrane with a CAAX motif [21,24]. We monitored the variations in fluorescence, both in the soma (figure 1*a*,*b*) and in the main process of each transfected neuron, between 10 and 20 µm from the soma (figure 1*c*–*e*). This region of the DRG main neurite corresponds to the proximal segment, equivalent to the axon initial segment, involved in AP generation [25]. In response to a single AP, evoked by a 1 ms current pulse, we recorded an intracellular Ca^2+^ rise that peaked within 300 ms in both locations (see figure 1*d* for time course in processes). In the cell bodies, the average peak response was reduced by 36% in α_2_δ-1 KO DRG neurons, compared with their WT counterparts (figure 1*b*), which is in good agreement with the previously observed approximately 30% reduction of VGCC current density [19]. Interestingly, the average Ca^2+^ peak in response to a single AP was reduced to a greater extent (63%) in the processes than in the cell bodies of neurons from α_2_δ-1 KO mice, compared with WT mice (figure 1*e*).

### (b) Comparison of properties of the first AP evoked by rheobasic current in WT and α_2_δ-1 KO DRG neurons

After determining that cultured DRG neurons from α_2_δ-1 KO mice showed a lower intracellular Ca^2+^ response to a single AP, we next investigated whether the absence of α_2_δ-1 would also affect the shape of the AP. The α_2_δ-1 subunit is associated with high-voltage-activated VGCCs, which, as a result of their kinetic properties, can be activated on the falling phase of the AP.

First we investigated the basic electrophysiological properties of the DRG neurons. The RMP was the same in WT and α_2_δ-1 KO DRG neurons (table 1) and was in agreement with values reported earlier [26,27]. Only cells with an RMP more negative than −40 mV were used in experiments. The *C*_m_ was smaller in male but not female α_2_δ-1 KO, compared to WT DRG neurons (table 1). The *R*_in_, measured in response to a −10 pA step, was also not different between WT and α_2_δ-1 KO DRG neurons (table 1).

**Table 1.** Basic electrophysiological parameters of WT and α_2_δ-1 KO DRG neurons. The C_m_ of the neurons reflects the small size of the somata after the outgrowth of neurites. *n* = number of DRG neurons examined.

	WT		α_2_δ-1 KO	
RMP (mV)	−52.5 ± 1.4 mV (*n* = 36)		−54.3 ± 1.3 mV (*n* = 43)	
*C*_m_ (pF)	17.3 ± 0.8 (male, *n* = 55) 13.9 ± 0.8 (female, *n* = 13)		15.0 ± 0.8*(male, *n* = 54) 12.4 ± 0.9 (female, *n* = 17)	
HP (mV)	−60	−70	−60	−70
*R*_in_ GΩ (*n*)	1.45 ± 0.23 (32)	1.66 ± 0.24 (33)	1.32 ± 0.14 (49)	1.78 ± 0.20 (43)

*p < 0.05 compared to WT.

**Figure 2.**
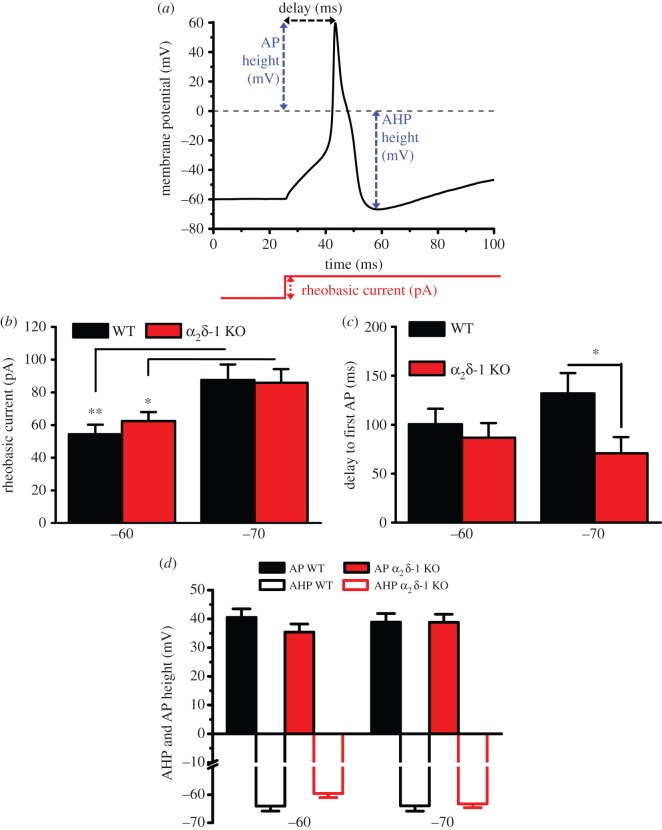
Properties of the first AP evoked by rheobasic current pulses in α_2_δ-1 KO and WT DRG neurons. (*a*) Description of measurements made on first AP. The red line shows the current stimulation. AHP, after-hyperpolarization. (*b*) Rheobasic current is the current evoking the first AP. Data shown are mean + s.e.m. for *n* = 32, 49 at −60 mV and 33, 43 at −70 mV for WT (black bars) and α_2_δ-1 KO (red bars) DRG neurons, respectively. *p < 0.05, **p < 0.01, Student's *t*-test. (*c*) Time delay to AP firing at rheobasic current, measured from the beginning of test current pulse to the peak of the first AP. *p < 0.05, Student's *t*-test. (*d*) Peak (mV) of AP (solid bars) and AHP (open bars) for WT (black bars) and α_2_δ-1 KO (red bars). For panels (*c,d*), data are mean + s.e.m. for the same cells shown in (*b*).

In order to test the impact of α_2_δ-1 KO on the shape of individual APs, we examined the first AP evoked by rheobasic current. In this way, we tried to minimize artefacts resulting from the response to multiple firing, such as the state of activation and inactivation of ion channels or from injection of current much larger than needed to pass the threshold of AP initiation. Figure 2 summarizes the basic properties of the first AP evoked by rheobasic current at two HPs, as described in figure 2*a*. As expected, there was an increase of the rheobasic current needed to trigger an AP in DRG neurons recorded from −70 mV compared to −60 mV HP; however, the genotype did not affect the rheobasic current (figure 2*b*). KO of α_2_δ-1 also had no effect on the time from the start of the current pulse to the peak of the first AP in cells recorded from −60 mV (figure 2*c*). However, when cells were recorded from −70 mV HP, the mean time delay to the first AP was shorter in α_2_δ-1 KO compared with WT DRG neurons (figure 2*c*). By contrast, α_2_δ-1 KO had no effect on the AP peak voltage (figure 2*d*), or on the peak of the after-hyperpolarization (AHP) (figure 2*d*), irrespective of the HP from which cells were recorded.

**Figure 3.**
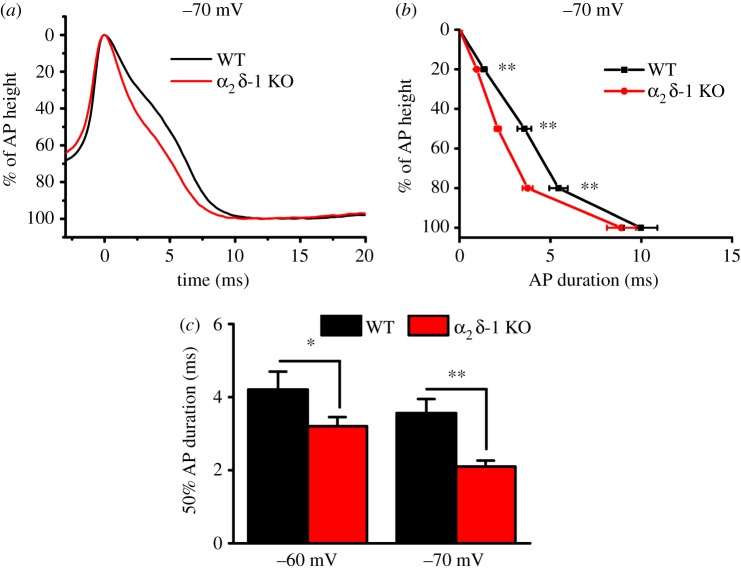
Comparison of AP properties in α_2_δ-1 KO and WT DRG neurons. (*a*) Example traces representing typical AP recordings in WT (black) and α_2_δ-1 KO (red) DRG neurons from −70 mV HP. (*b*) Duration of first AP from peak of the AP (0%) to peak of the AHP (100%), evoked by rheobasic current, recorded from −70 mV HP, in WT (black squares, *n* = 33) and α_2_δ-1 KO (red circles, *n* = 43) DRG neurons. AP duration was significantly shortened at 20%, 50% and 80%, but not 100% of AP duration. **p < 0.01 α_2_δ-1 KO versus WT, Student's *t*-test. (*c*) AP duration at 50% of AP height, recorded from −60 mV (left) and −70 mV (right) HP in WT (black bars) and α_2_δ-1 KO (red bars) DRG neurons. *p < 0.05, **p < 0.01, Student's *t*-test.

To test whether α_2_δ-1 KO affects the duration of the AP, we examined the width of the first AP evoked by rheobasic current, from 0% (the peak of AP) to 100% (the peak of AHP), and measured the standardized AP width at 20–100% after the peak of the AP, for the same cells described in figure 2. In α_2_δ-1 KO DRG neurons, there was a consistent decrease in the AP width (figure 3*a*), at 20, 50 and 80%, compared with WT DRG neurons at −70 mV HP (figure 3*b*,*c*), whereas at −60 mV this difference was only observed at 50% AP width (figure 3*c*). By contrast, the 100% duration of the AP was not affected, being 11.14 ± 0.98 ms and 9.96 ± 0.92 ms in WT DRG neurons, compared to 11.45 ± 0.72 ms and 8.90 ± 0.80 ms in α_2_δ-1 KO DRG neurons, at −60 and −70 mV HPs, respectively.

### (c) Comparison of multiple action potential firing evoked by a 400 ms current pulses in WT and α_2_δ-1 KO DRG neurons

**Figure 4.**
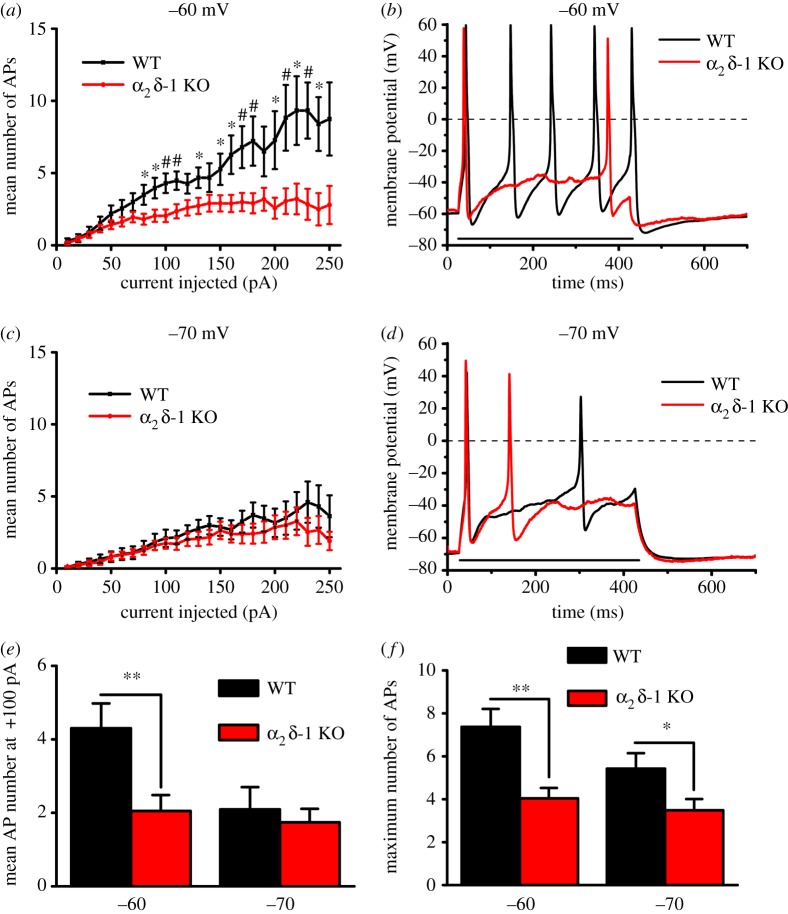
Number of APs evoked by current pulses in α_2_δ-1 KO and WT DRG neurons. (*a*) APs recorded from −60 mV HP were evoked with 400 ms current pulses, increasing every +10 pA. The mean (±s.e.m.) number of APs is shown at each current pulse for the WT (black squares, *n* = 32) and α_2_δ-1 KO (red circles, *n* = 49) DRG neurons. Differences at each step were examined with Student's *t*-test; *p < 0.05, ^#^*p* < 0.01. The slopes of the relationships were also fitted by linear regression up to +200 pA, being 0.038 ± 0.0007 and 0.016 ± 0.001 for WT and α_2_δ-1 KO cells, respectively (*p* < 0.001, *F*-test). (*b*) Examples of AP firing during injection of 400 ms, +100 pA current (represented by the horizontal black line) in WT (black trace) and α_2_δ-1 KO (red trace) DRG neurons from −60 mV HP. (*c*) APs recorded from −70 mV HP were evoked as in (*a*). The mean (±s.e.m.) number of APs at each current pulse for the WT (black squares and lines, *n* = 33) and α_2_δ-1 KO (red circles and lines, *n* = 43) DRG neurons is shown. The slope of the relationships fit up to +200 pA by linear regression was 0.018 ± 0.001 and 0.013 ± 0.001 for WT and α_2_δ-1 KO cells, respectively (*p* < 0.001, *F*-test). (*d*) Examples of AP firing during injection of 400 ms +100 pA current (represented by the horizontal black line) in WT (black trace) and α_2_δ-1 KO (red trace) DRG neurons from −70 mV HP. (*e*) Comparison of the mean ± s.e.m. firing frequency evoked with +100 pA current pulse for WT (*n* = 30, 31) and α_2_δ-1 KO (*n* = 39, 39) DRG neurons at −60 and −70 mV HP, respectively. (*f*) Mean + s.e.m. of the maximum firing frequency, irrespective of current injected, recorded in each cell in WT (*n* = 32, 33) and α_2_δ-1 KO (*n* = 49, 43) DRG neurons at −60 and −70 mV respectively. For (*e*,*f*), *p < 0.05, **p < 0.01, Student's *t*-test.

We then assessed whether there were changes in the firing properties of α_2_δ-1 KO compared to WT DRG neurons. To assess neuronal excitability, biased current was injected to bring the cell membrane to a HP of −60 or −70 mV, the first being close to the DRG neuron RMP, whereas the second was chosen to be outside the steady-state window current region of T-type channels [28]. Next, a set of 400 ms-long current pulses, increasing every 10 pA, was applied to estimate cell excitability induced by depolarization. These increasing current pulses elicited a robust increase in AP number (figure 4*a*–*d*), which was approximately linearly dependent on injected current, for WT DRG neurons, with a greater slope at −60 mV than at −70 mV HP (figure 4*a*,*c*). At −60 mV HP, the number of elicited APs was reduced in α_2_δ-1 KO compared with WT DRG neurons (figure 4*a*,*b*). By contrast, when cells were maintained at −70 mV HP, increasing positive current pulses elicited only a moderate response that was not different between the WT and α_2_δ-1 KO groups (figure 4*c*,*d*). A direct comparison between the mean responses to a +100 pA current pulse, from the two HPs, is shown in figure 4*e*. However, when the maximum number of APs was determined for each cell, irrespective of current injected, WT DRG neurons had a higher maximum AP firing frequency than α_2_δ-1 KO DRG neurons, at both −60 and −70 mV HPs (figure 4*f*). Thus, overall, α_2_δ-1 KO significantly reduced the excitability properties of DRG neurons, particularly when recorded from −60 mV HP.

### (d) Does the effect of α_2_δ-1 KO involve activation of K^+^ channels?

In previous studies in hippocampal neurons, it has been suggested that the possible mechanism of overexpressed α_2_δ-1-induced changes in neuronal excitability and AP waveform may involve activation of K^+^ channels [13,14]. Thus, we explored here whether the changes in DRG neuron excitability and AP waveform involved activation of Ca^2+^-activated K^+^ channels. Two K^+^ channels were selected for this study as candidates involved in the change in AP waveform and neuronal excitability in α_2_δ-1 KO mice. Large conductance calcium-activated potassium (BK) channels, blocked by iberiotoxin, directly interact with VGCCs [29]. Small conductance potassium (SK) channels, blocked by apamin, are associated with calmodulin, which acts as a Ca^2+^ sensor [30].

#### (i) Effect of iberiotoxin

**Figure 5.**
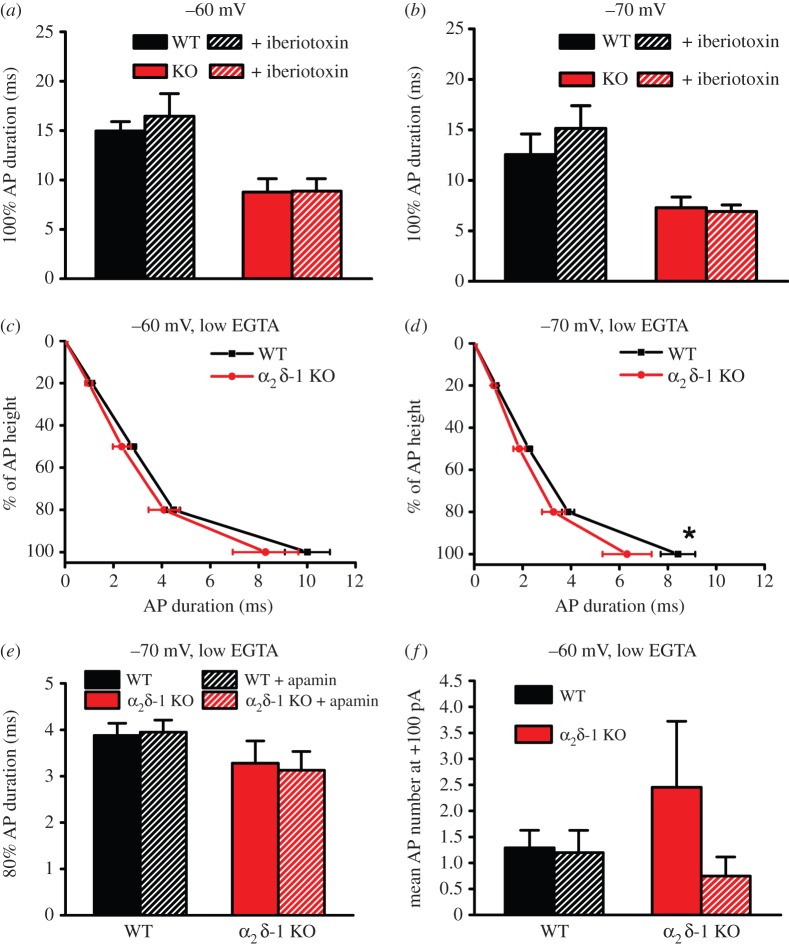
Effect of BK and SK channel blockers and reduced intracellular Ca^2+^ chelation on AP duration. (*a*,*b*) Duration of 100% AP height in WT (black bars, *n* = 11 at −60 mV and *n* = 10 at −70 mV) and α_2_δ-1 KO (red bars, *n* = 10 for both HPs) DRG neurons, and lack of effect of iberiotoxin (0.1 µM, hatched bars), from HP of −60 mV (*a*) and −70 mV (*b*). (*c*,*d*) Comparison of AP duration between WT (*n* = 21, 24; black squares) and α_2_δ-1 KO (*n* = 10, 10; red circles) DRG neurons using the low intracellular Ca^2+^-buffering intracellular solution, at −60 mV (*c*) and −70 mV (*d*) HP. *p < 0.05, 2-way ANOVA followed by Bonferroni's *post hoc* test. (*e*) 80% AP duration (mean + s.e.m.) for the WT (black bars) and α_2_δ-1 KO (red bars) cells shown in (*d*), prior to and during apamin (0.5 µM, hatched bars) application, at −70 mV HP. The effect of apamin was determined for each cell. (*f*) Comparison of the firing frequency (mean + s.e.m.) evoked with +100 pA 400 ms current pulse for WT (black bars; *n* = 24) and α_2_δ-1 KO (red bars, *n* = 11) DRG neurons at −60 mV using the low intracellular Ca^2+^-buffering intracellular solution. The lack of effect of apamin is shown in the hatched bars for WT (black bars; *n* = 20) and α_2_δ-1 KO (red bars, *n* = 8).

Since BK channels are located in close proximity to VGCCs, they are very sensitive to changes in Ca^2+^ concentration as a consequence of VGCC activation. Thus, during the falling phase of the AP, BK channels open and close rapidly in response to Ca^2+^ entry, in synergy with their response to depolarization [31]. In this way, BK channels are part of the mechanism of tight control of AP duration in many excitable cells. However, the application of iberiotoxin (0.1 µM) had no effect on AP duration in WT or α_2_δ-1 KO DRG neurons, recorded from either −60 mV (figure 5*a*) or −70 mV (figure 5*b*). For this reason, we also examined the involvement of SK channels [30] in AP duration.

#### (ii) Effect of low intracellular Ca^2+^ buffering and apamin

Unlike BK channels, SK channels do not interact directly with VGCCs. Thus, to activate SK channels, Ca^2+^ must diffuse to bind with SK-associated calmodulin. In order to enable SK channel activation, the internal EGTA concentration was reduced, while maintaining the internal free [Ca^2+^]. Interestingly, under these conditions, the AP waveform in WT DRG neurons was reduced in width. For example, from −70 mV HP, the 50% AP width was 3.56 ± 0.38 ms under control conditions (figure 3*b*) and 2.25 ± 0.14 ms (figure 5*d*) in the low intracellular [Ca^2+^] buffering condition (*p* < 0.01, Student's *t*-test). Furthermore, in the low intracellular [Ca^2+^] buffering condition, the AP waveform was not significantly different between WT and α_2_δ-1 KO DRG neurons, at −60 mV (figure 5*c*), and only reduced at 100% AP duration in α_2_δ-1 KO DRG neurons at −70 mV (figure 5*d*). However, application of apamin (0.5 µM) produced no significant effect on AP duration in WT or α_2_δ-1 KO DRG neurons (see, for example, 80% AP duration at −70 mV, figure 5*e*).

Furthermore, in the low intracellular [Ca^2+^] buffering condition, AP firing frequency was markedly reduced in WT DRG neurons, compared with that in the normal intracellular Ca^2+^ conditions (1.29 ± 0.34, figure 5*f*, compared to 4.30 ± 0.67 APs during the 400 ms stimulation from −60 mV HP, figure 4*e*; *p* *<* 0.001, Student's *t*-test). Finally, AP firing frequency was not different between WT and α_2_δ-1 KO DRG neurons in the low intracellular [Ca^2+^] buffering condition, and the application of apamin had no effect (figure 5*f*).

## 4. Discussion

In this study, the main findings are that in cultured DRG neurons from α_2_δ-1 KO mice, there is reduced Ca^2+^ entry on single AP stimulation, reduced AP duration and reduced firing frequency to a 400 ms stimulation, compared in all cases with DRG neurons from WT littermates, consistent with the expected roles of VGCCs in these events. Furthermore, lower intracellular Ca^2+^ buffering also resulted in reduced delay to AP firing, reduced AP duration, and a lower frequency of AP firing in WT DRG neurons, mimicking the effect of α2δ-1 KO.

Our finding that deletion of the α_2_δ-1 gene in mice significantly reduced the rise in intracellular Ca^2+^ in response to a single AP stimulation in DRG neuron somata was predicted from our previous study, showing that calcium channel currents (both N-type and residual non-N-type) were reduced in α_2_δ-1 KO DRG neurons [19]. It is of interest that there was a greater effect of α_2_δ-1 KO on the intracellular Ca^2+^ rise induced by a single AP in the proximal segment of the main axon than in the cell body, pointing to an important role of α_2_δ-1 in VGCC trafficking into the processes, and possibly reflecting a difference in AP generation. Furthermore, the reduced AP delay found in α_2_δ-1 KO DRGs may reflect different locations of AP initiation in the two genotypes. Thus in α_2_δ-1 KO DRGs, AP initiation may be in the soma rather than the proximal segment.

In agreement with the Ca^2+^ imaging results, this study identified a reduction in the duration of single APs in α_2_δ-1 KO DRG neurons, compared with WT neurons. The 50% AP duration was reduced, and the effect of α_2_δ-1 was stronger when the HP was −70 mV compared to −60 mV, possibly because more VGCCs are normally available from the more negative HP in WT DRG neurons. The AP shortening in α_2_δ-1 KO DRG neurons would be consistent with reduced Ca^2+^ entry resulting from a single AP. There is potential for a dual effect of VGCC activation on the AP waveform, as Ca^2+^ entry during the AP results in the appearance of a ‘hump’ on the repolarization phase of the AP, but will also activate Ca^2+^-dependent processes involved in AP termination [1], including activation of K^+^ channels [30,32,33] and Ca^2+^-dependent inactivation of VGCCs [34,35]. For example, the effect of reduced inactivation of Ca_V_1.2 on the cardiac AP is predicted from computer modelling to result in a marked AP prolongation [36]. In contrast, in DRG neurons, application of a cocktail of calcium channel blockers also prolonged AP duration [37].

In previous studies, overexpression of α_2_δ-1 resulted in shortening of the AP duration, both in DRG neurons [13] and in hippocampal neuron synaptic terminals, the latter measured using a fluorescent membrane voltage sensor [14]. By contrast, peripheral axotomy and spinal nerve ligation in rats were found to result in increased AP duration in DRG neurons [37,38], accompanied by reduced calcium currents in medium-sized DRG neurons [37], despite upregulation of α_2_δ-1 following peripheral sensory nerve injury [15]. However, nerve injury models result in up- or downregulation of many transcripts [39,40], which may also impact on AP duration, calcium current amplitudes and altered channel trafficking. Furthermore, genetic manipulation of mice may have secondary effects on expression of other genes, although we have found that α_2_δ-2 and α_2_δ-3 are not upregulated in compensation for the loss of α_2_δ-1 in the mice studied here [19].

In this study, reduced buffering of intracellular [Ca^2+^] in the recording pipette had a pronounced effect, alone, to reduce AP duration, possibly via the processes described above. Furthermore, low intracellular [Ca^2+^] buffering occluded the reduction in AP duration present in α_2_δ-1 KO DRG neurons. By contrast, applying blockers of Ca^2+^-activated BK and SK channels had no consistent effect on AP duration. In a previous study, we identified a role for α_2_δ-1 in co-localizing VGCCs with the endoplasmic reticulum and mitochondrial pathways involved in the control of buffering the Ca^2+^ rise through N-type VGCCs [41]; thus a modification of VGCC localization in α_2_δ-1 KO DRG neurons might alter their ability to buffer Ca^2+^ entering through N-type VGCCs.

We then examined the firing frequency of isolated DRG neurons, and found a lower firing frequency in α_2_δ-1 KO compared with WT DRG neurons, particularly when cells were maintained close to their RMP, at –60 mV. By contrast, from an HP of −70 mV, the firing frequency to increasing depolarizations was markedly reduced, and there was little effect of α_2_δ-1 KO. Furthermore, we also found that low intracellular Ca^2+^ buffering reduced AP firing frequency in WT DRG neurons, whereas it had no effect in α_2_δ-1 KO DRG neurons.

A difference of 10 mV in the HP, between −60 and −70 mV, can produce a significant influence on the opening of various subtypes of VGCCs and other channels, such as hyperpolarization-activated (HCN) channels that open upon hyperpolarization negative to −50 mV, and may result in reduced AP firing in response to current injection [42]. Furthermore, at −70 mV, most VGCCs are closed and available, so the inflow of Ca^2+^ is restricted to when the APs are activated. By contrast, at −60 mV, some VGCCs, particularly the low-voltage-activated Ca_V_3 channels, have a window current [28]. Ca_V_1.3 also begins to activate below −60 mV in 2 mM Ca^2+^ [43], and shows incomplete steady-state inactivation [44], which may also result in a Ca^2+^ window current, depending on the splice variant expressed [45]. However, in mouse chromaffin cells, knockout of Ca_V_1.3 actually leads to a biphasic effect on AP firing, a reduction in frequency at small depolarizing currents and an increase in frequency at large depolarizing current, because of the loss of SK activation [46]. It is difficult to examine the influence of L-type channels on the AP waveform, as 1,4-dihypropyridine calcium antagonists, such as nifedipine, do not inhibit L-type channels (Ca_V_1.2 or 1.3) opened by single APs [43], and no drugs discriminate adequately between Ca_V_1.2 and Ca_V_1.3 [47].

In summary, we have previously found that α_2_δ-1 KO leads to a reduction of Ca^2+^ influx through VGCCs in DRG neurons [19]. Our main findings here are that α_2_δ-1 KO leads to reduced Ca^2+^ entry on single AP stimulation in DRG processes, as well as reduced AP duration and reduced firing frequency to a 400 ms stimulation in α_2_δ-1 KO neurons, consistent with the expected role of VGCCs in these events. Furthermore, lower intracellular Ca^2+^ buffering also reduced AP duration, and lowered the frequency of AP firing in WT DRG neurons, mimicking the effect of α_2_δ-1 KO. By contrast, we did not obtain any consistent evidence for the involvement of Ca^2+^-activation of BK and SK channels in these events. Future research could examine the role of other ion channels involved in AP generation and termination [42,48], to pin-point the molecular mechanism for the effects of α_2_δ-1 KO described here.

## Data accessibility

The α_2_δ-1 knockout mice strain (*cacna2d1*^+/−^) have been deposited in the EMMA repository, MRC Harwell.
